# Dopamine Receptor Autoantibodies Correlate with Symptoms in Sydenham's Chorea

**DOI:** 10.1371/journal.pone.0073516

**Published:** 2013-09-20

**Authors:** Hilla Ben-Pazi, Julie A. Stoner, Madeleine W. Cunningham

**Affiliations:** 1 Pediatric Movement Disorders, Neuropediatric Unit, Shaare Zedek Medical Center, Jerusalem, Israel; 2 Department of Biostatistics and Epidemiology, University of Oklahoma Health Sciences Center, Oklahoma City, Oklahoma, United States of America; 3 Department of Microbiology and Immunology, University of Oklahoma Health Sciences Center, Oklahoma City, Oklahoma, United States of America; University of Utah School of Medicine, United States of America

## Abstract

**Background:**

Sydenham chorea (SC), a neuropsychiatric sequela of group-A streptococcal infection, is associated with basal ganglia autoantibodies. Although autoantibodies have been proposed in neuropsychiatric disorders, little evidence has been shown to link autoimmunity and clinical symptoms. We hypothesized that dopamine receptor-autoantibody interactions may be the basis of neuropsychiatric symptoms in SC.

**Methods:**

Sera from 22 children with SC (age 10.7±4.5 years) and 22 age-matched controls were studied. Clinical neuropsychiatric symptoms were measured in SC at sample collection using the UFMG-Sydenham's-Chorea-Rating-Scale (USCRS). Anti-dopamine D1 receptor (D1R) and anti-dopamine D2 receptor (D2R) autoantibodies were measured by the enzyme linked immunosorbent assay (ELISA) and were correlated with clinical symptoms.

**Results:**

Anti-D1R and anti-D2R autoantibodies were significantly higher in SC compared to controls (n = 44; p = 0.010 and p = 0.017, respectively). We found that the ratio (anti-D2R/D1R) of the two anti-dopaminergic receptor antibodies correlated with neuropsychiatric symptoms as determined by USCRS measurements (n = 18; r = 0.53, p = 0.024). In addition, anti-D2R titers correlated with antistreptolysin-O titers (n = 43; r = 0.49, p = 0.0008).

**Interpretation:**

Our report linked, for the first time, autoimmunity with neuropsychiatric symptoms. The significant correlation was found using ratios of autoantibodies against dopamine receptors (anti-D2R/D1R) rather than the absolute elevated individual anti-D1R or anti-D2R titers. We suggest that autoantibodies may lead to a receptor imbalance and induce greater sensitivity to dopamine signaling potentially leading to neuropsychiatric symptoms in SC. Our novel findings suggesting altered balance in the dopaminergic system may provide a new approach in understanding autoimmune neuropsychiatric disorders with possible implications for diagnosis and treatment.

## Introduction

Sydenham's chorea (SC) is a disabling pediatric hyperkinetic and neuropsychiatric disorder following streptococcal infection. Its clinical characteristics encompass both motor and behavioral symptoms, manifesting as emotional lability, hyperactivity, irritability, distractibility, and obsessive-compulsive symptoms predating the chorea which may have a prolonged course leading to significant functional impairment [1]. SC pathogenesis has been considered to be an autoantibody-mediated basal ganglia dysfunction since antibodies derived from children with SC demonstrate an affinity to basal ganglia components [2] and anti-inflammatory treatments such as steroids, plasmapheresis and intravenous immunoglobulin treatment are effective [3]. However, it is not known whether SC-associated autoantibodies induce clinical symptoms or if they are merely biomarkers secondary to the inflammatory process in the basal ganglia. Clearly, the idea of dopamine involvement in the disease is relevant since it is thought to fulfill an important role in the pathophysiology of chorea [4], and the symptomatic treatment in SC relies on the use of anti-dopaminergic drugs.

Autoantibodies, such as anti-lysoganglioside (LGN) GM1 [5] and anti-beta tubulin [6] described in SC may be involved indirectly in dopaminergic pathways. Recently, a rat model exposed to streptococcal antigens exhibited motor and behavioral symptoms as well as elevated anti-D1R and anti-D2R antibodies [7] and antibodies to surface D2R were found in patients with SC [8]. Because of the central role of dopamine in SC, we investigated whether autoantibodies that might affect dopaminergic neurotransmission, such as anti-D1R and anti-D2R antibodies, were present in children with active SC and if they correlated with non-motor and motor symptoms. Most importantly, our study is one of the first to directly link autoimmunity against dopamine receptors and clinical neuropsychiatric symptoms in humans.

## Materials and Methods

### Participants and Sera

Sera were collected from 22 children and young adults with symptomatic SC (mean age 10.7±4.5 (SD) years; 16 females; 15 Ashkenazi ethnic background) from the pediatric movement disorders clinic at Shaare Zedek Medical Center and from 22 age-matched controls (age 10.1±4.1 years; 11 females; 12 Ashkenazi; Table 1). There was no significant difference between the groups in terms of age (Wilcoxon rank sum test, p = 0.81), gender (Chi-square test, p = 0.12) or ethnicity (Chi-square test, p = 0.35). Eighteen children in the study group had an acute course, 3 recurrent and 1 persistent SC (>12 months). Clinical and laboratory data were available for all children with SC; 18/22 were assessed systematically using the UFMG Sydenham's Chorea Rating Scale (USCRS) [9] a validated systematic rating of motor and non-motor symptoms of SC within a week of blood tests (by Dr Ben-Pazi). USCRS could be further divided into non-motor (sum of items 1–6) and motor (sum of items 7–21) scores. Most participants were treated with penicillin (7 orally (33%) and 9 (43%) intramuscularly among the 21 with known treatment information). Chorea was treated in 9/21 (three with valproic acid, three with prednisone, two by neuroleptics and one with carmazapine) during the time of the study. Children without acute neurological illness treated in the hospital were used as controls. Control sera were collected (taken during their routine blood tests) from 14 children treated in the day care unit, 5 in emergency room, and 3 from outpatient clinics were collected during their routine blood tests. Controls with elevated ASO and streptococcal or other infections were not excluded. The study was approved by Shaare Zedek Medical Center Helsinki committee and University of Oklahoma Health Sciences Center Internal Review Boards. Written informed consents were obtained for all participants according to the ethics committees' approval. Consent forms were signed by parents or legal guardians on the behalf of children younger than 18 years. Individuals over age 18 years signed written consent forms themselves.

**Table 1:** Clinical characteristics of children with Sydenham's chorea.

Age, Gen	Dx	Duration	Carditis	Arthritis	USCRS	Non-motor	Motor
4.5, F	Ac SC	0.25	M&A endocarditis	N	22	3	19
6, M	Ac SC	3	mild MR	N	19	2	17
6, F	Ac SC	1	mod MR, mild AR	Y	12	1	11
6, F	Ac SC	0.5	MR AR	Y	21	3	18
6, M	Ac SC	0.5	later developed MR	N	X	X	X
7, F	Ac SC	2	mod MR, mild AR, mild-mod TR	N	X	X	X
7, F	Ac SC	0.5	mild-mod MR, mild AR	N	6.5	0	6.5
8, F	Ac SC	2	mild-mod MR	N	27	2	25
9, F	Ac SC	4	mild MR, Trace AR	N	15	2	13
10, F	Ac SC	2	not performed	Y	34	17	17
12, F	Ac SC	1.5	mod- severe MR, mild AR	Y	15	0	15
12.5, M	Ac SC	1	mod severe MR	N	3.5	0	3.5
13, F	Ac SC	0.5	mild mod AR/MR	Y	12	2	9.5
13.5, F	Ac SC	4.5	trace AR-> Mild AR	N	3	1	2
14, F	Ac SC	0.5	MR, Mod AR	Y	27	6	21
15, F	Ac SC	0.1	mild MR and AR	N	16	0	16
17, M	Ac SC	0.5	mild-mod MR, mild AR	Y	19	4	15
19, F	Ac SC	0.5	mild AR	N	X	X	X
8, F	RSC	X	mod MR	N	X	X	X
9, M	PSC	36	mild MR	N	36	5	31
13, F	RSC	0.5	mod MR	N	49	6	43
20, M	RSC	70	mild AR, mild MR	N	9.5	0	9.5

*Gen = Gender; Dx =  Diagnosis; Duration =  time in months since initial symptoms to clinical and serologic examination. Ac SC =  Acute SC, PSC =  Persistent SC, RSC =  Recurrent SC; M&A =  mitral and aortic; MR- mitral regurgitation; TR- tricuspid regurgitation;* X = No data.

### Laboratory testing

All samples were coded and researchers were blinded to the diagnosis and the identity of all samples. Enzyme Linked Immunosorbent Assay (ELISA) was performed in 96-well Immunolon microtiter plates (Fisher Thermo Scientific, Rochester, NY, USA). Fifty μl of antigen (10 μg/ml human dopamine D1R or D2RL, Perkin Elmer (Waltham, MA, USA), 10 μg/ml beta-tubulin(MP Biomedicals, Solon, OH, USA), 20 μg/ml lysoganglioside-GM1(LGN)) (from bovine brain, Sigma Aldrich Corporation, St Louis, MO, USA) and 10 ug/ml bovine serum albumin (BSA) (Fisher Thermo Scientific, Rochester, NY, USA). We used human dopamine D1R or D2R-Long (L) (which are single chain) from Perkin Elmer for the detection of the anti-D1R and anti-D2R. All antigens were coated onto plates overnight at 4°C. Plates were then washed 3X with 0.1% Tween (Fisher Thermo Scientific, Rochester, NY, USA) in phosphate buffered saline (PBS) pH 7.2. Plates were blocked with 1% bovine serum albumin (BSA (Fisher Thermo Scientific, Rochester, NY, USA), washed 3X and incubated at 37°C for 30 minutes and overnight at 4°C with serum diluted 1/100 in 1% BSA and titrated 2-fold to obtain a titration of the sera. After washing, plates were incubated with secondary antibody affinity purified anti-human IgG gamma chain specific (Jackson Laboratories, Inc) diluted 1:1000 in 1% BSA, washed again, and developed with *p*-nitrophenylphosphate (Sigma Aldrich Corporation, St. Louis, MO, USA). Optical density values were measured at 405 nm on an automated Dynatech Microplate Reader and corrected by blanks (antigen without serum). Assays were performed in duplicate. Titers represent the serum dilution reading at optical density of 0.1 at 405 nm after two hours of incubation.

### Data analysis

The outcome measures were the antibody titer levels at two hours for anti-D1R anti-D2R, anti-tubulin, anti-LGN and antistreptolysin-O (ASLO) antibodies. Data were analyzed using SAS (SAS System for Windows, ver. 9.1, SAS Institute Inc., Cary, NC). The distributions of continuous demographic factors and antibody titer levels were compared between cases and controls using a non-parametric Wilcoxon rank sum test. A Chi-square test, or Fisher's exact test when more than 20% of cross-tabulation expected frequency counts were less than 5, was used to compare proportions between cases and controls. A Kruskal-Wallis test was used to compare the distribution of antibody titer levels among three groups defined by time since diagnosis. The correlation between pairs of continuous symptom scores and antibody titer levels was summarized using Spearman's rank correlation coefficient. Summary statistics results are reported as the mean ± standard deviation or the median [25^th^ percentile, 75^th^ percentile] where the 25^th^ percentile is the value at which 25% of the data lie below, and the 75^th^ percentile is the value at which 75% of the data lie below. For exploratory purposes and to identify potential confounding or modifying factors, analysis of the correlation between symptom scores and antibody titer levels, as well as analysis of the difference in titer levels between cases and controls, was performed after stratifying by disease duration, gender and ethnic group. For all statistical tests, the level of significance was set at a two-sided 0.05 alpha level.

## Results

### Anti-D1R and anti-D2R IgG titers were significantly elevated in Sydenham chorea (SC)

**Figure 1:**
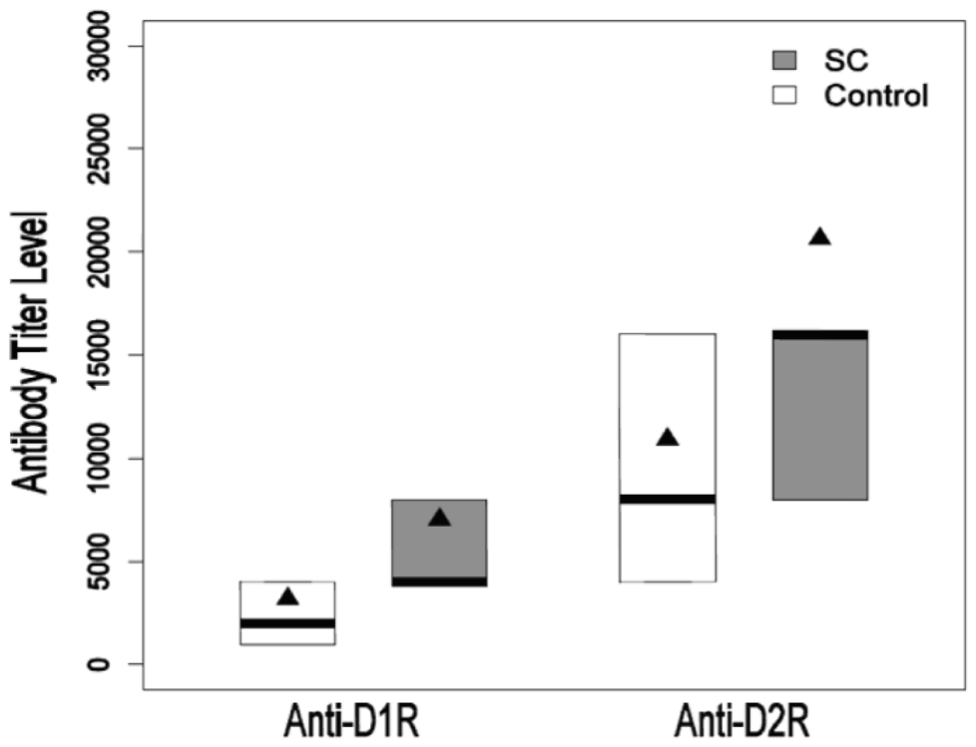
Distribution of Anti-D1R and Anti-D2R IgG antibody titers of Sydenham's chorea patients and controls. Box plots present Sydenham's chorea (SC; grey) and control (white) group anti-D1R and anti-D2R titers between 25th and 75th percentiles with elevated mean (triangle) and median (bar) titers in SC. Median and percentiles may share same values due to multiple measurements.

In children with SC, we found significantly elevated anti-D1 receptor (D1R) (*P* = 0.010) and anti-D2 receptor (D2R) (*P* = 0.017) IgG antibody titers compared with age matched controls by the Wilcoxon rank sum test (Figure 1). Statistical analysis revealed a median titer of anti-D1R antibody as 4,000 [25^th^ percentile (p25) – 75^th^ percentile (p75)  = 4,000–8,000] versus control median value of 2,000 [p25–p75 = 1,000–4,000]. The mean anti-D1R IgG titer was 7045 which was approximately 3 standard deviations (2.7) above the control mean. The anti-D2R IgG antibody had a median titer of 16,000 [p25–p75 = 8000–16,000] which was significantly elevated compared with the median of 8,000 [p25–p75 = 4,000–16,000] of the controls (Figure 1). The mean anti-D2R IgG titer was 20,636 which was approximately 2 standard deviations (1.9) above the control mean. Anti-D1R and anti-D2R did not correlate with each other in the study sample (n = 44, r = 0.24, p = 0.11) nor among SC patients when considered separately (n = 22, r = −0.04, p = 0.88).

Anti-tubulin and anti-LGN were not significantly higher among SC patients compared to controls (p = 0.60 and p = 0.66, respectively). This result is in contrast to previous findings and is due to our selection of a different control population which had an overall 3X higher ASLO titer than our previous controls. In the current setting, controls differ in ethnicity and ASLO titer. In addition, antibodies against bovine serum albumin (BSA), a non-neuronal antigen, were not significantly higher among SC patients (median titer [p25–p75] = 1000) compared to controls (median titer [p25–p75] = 500; p = 0.09). Therefore, antibodies in SC specifically recognized the dopamine receptors D1R and D2R with highly elevated IgG titers as shown (See figure 1) which were far above the titers observed for BSA, lysoganglioside and tubulin.

### Anti-D2R/D1R ratio correlated with clinical symptoms

**Figure 2:**
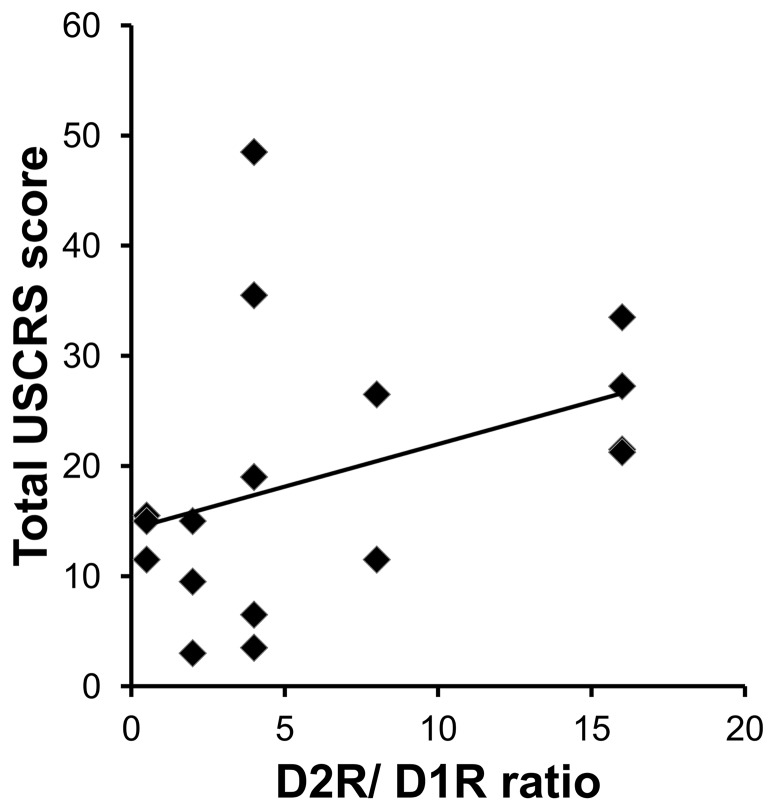
Dopamine receptor antibody ratio (D2R/D1R) correlates with neuropsychiatric symptoms (USCRS score). Autoantibody ratio against dopaminergic receptors correlated with the clinical motor and non-motor symptoms. Total USCRS ratings (*n* = 18, mean 19.1±11.9) correlated with anti-D2R/anti-D1R ratio (*r* = 0.53, *P* = 0.024) suggesting that the receptor imbalance may lead to dopaminergic hypersensitivity causing the clinical neuropsychiatric symptoms.

When we examined all of the cases with clinical score data (USCRS; n = 18), we found that the ratio between the levels of the two anti-dopaminergic receptor antibodies corresponded with neuropsychiatric symptoms. While anti-D2R/D1R ratio correlated with USCRS total score (r = 0.53, p = 0.024; Figure 2) other antibodies did not (ASLO: r = −0.2, p = 0.43). Non-motor symptoms had a higher correlation with antibody titer ratio (r = 0.59, p = 0.010) compared to the motor symptoms (r = 0.47, p = 0.050). Irritability, attention deficit, hyperactivity, walking, and tongue protrusion were the individual items most strongly correlated with the anti- D2R/D1R ratio (r>0.5 and p<0.03 for each; Table 2).

**Table 2:** Correlation of D2R/D1R ratio with specific symptoms in Sydenham chorea.

#	Symptoms in Sydenham chorea (USCRS)	D2R/D1R	
-------	-----------------------------------------	r*	p
**1**	Irritability	**0.80**	**<0.0001**
**2**	Attention deficit	**0.58**	**0.012**
**3**	Hyperactivity	**0.53**	**0.024**
**4**	Obsessions	0.23	0.36
**5**	Compulsions	0.34	0.17
**6**	Verbal fluency	−0.04	0.88
**7**	Speech	0.06	0.8
**8**	Chorea- history	0.17	0.49
**9**	Handwriting	0.34	0.18
**10**	Cutting food	0.36	0.14
**11**	Hygiene	−0.07	0.78
**12**	Dressing	0.11	0.65
**13**	Walking	**0.52**	**0.028**
**14**	Eye movements	−0.43	0.075
**15**	Dysarthria	0.33	0.18
**16**	Chorea- examination	0.17	0.49
**17**	Tongue protrusion	**0.54**	**0.02**
**18**	Finger tap	0.25	0.32
**19**	Leg	0.16	0.54
**20**	Muscle tone	0.43	0.076
**21**	Gait	0.35	0.15
**-----------**	**TOTAL**	**0.53**	**0.024**
**#1–6**	**Non-motor**	**0.59**	**0.01**
**#7–21**	**Motor**	**0.47**	**0.05**

* r  =  Spearman Correlation Coefficient and corresponding p-value.

Although each individual anti-D1R and anti-D2R antibody titer did not correlate significantly with total USCRS scores, or clinical motor or non-motor symptoms, the two together were associated with clinical disease.

### Anti-D2R titers correlated with clinical symptoms in untreated participants and with ASLO titers in all participants

As penicillin is the gold standard of treatment for SC, most (16/21 with known treatment status) SC was treated upon referral; however, there were 5 untreated cases in our study group. Among 4 subjects who had not been treated with penicillin for whom we had measured clinical scores, the total scores, the non-motor scores, and, the ratio of non-motor to motor scores were positively associated with Anti-D2R IgG antibody titers (r = 0.95, p = 0.05). In addition, in our combined SC group of all of the study participants (n = 44), we looked for associations between the titers of the different antibodies and found that anti-D2R was very positively correlated with ASLO (r = 0.49, p = 0.0008).

### Motor and non-motor symptoms positively correlated

**Figure 3:**
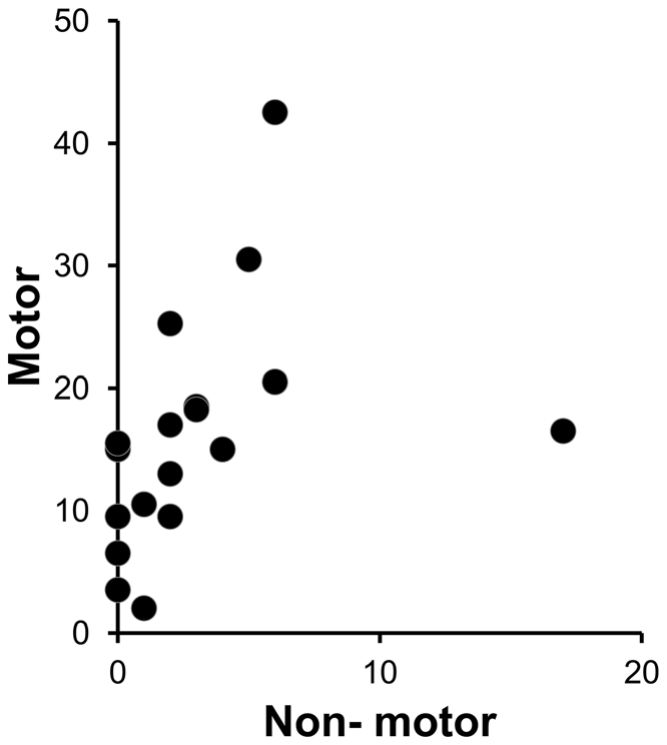
Motor scores correlate with non-motor scores of SC patients evaluated by USCRS. USCRS rates motor and non-motor symptoms on a 0–4 severity scale (0 = none to 4 =  severe). In our patients the sum of motor symptom (items 7–21) were closely correlated (r = 0.70, *P* = 0.0011) with the sum of non-motor items (1–6).

As expected, the non-motor symptoms rated by USCRS (sum of items 1–6) positively correlated with the motor symptoms (sum of items 7–21; n = 18, r = 0.70, p = 0.0011; Figure 3). The USCRS does not assess the change of symptoms over time; thus most of our SC patients reported non- motor and motor symptoms at the time of examination despite the fact that the behavioral changes were generally the first observed changes noted by the parents, preceding the chorea by 2 weeks (clinical observation, Dr Ben- Pazi).

## Discussion

Over the past years, clinical research groups have been searching for the targets of autoantibodies in neuropsychiatric illnesses including SC [5], [6] and related neurologic sequelae of group A streptococcal infections [10], [11], [12], [13]. Since SC is a well established neurologic manifestation of rheumatic fever, it may serve as a prototype for other streptococcal related neuropsychiatric disorders. It is possible that our data may advance the understanding of SC and other childhood neuropsychiatric disorders based on their relationship to anti-D1R and anti-D2R antibodies.

We found elevated anti-D1 and anti-D2 dopamine receptor antibodies in children with Sydenham chorea. The dopamine D1 and D2 receptors are heteromolecular complexes. Because D1R and D2R share amino acid homologies in their extracellular loops, they may also share epitopes, and the elevated antibodies directed at DR1 might represent antibody which binds with higher affinity to D2R. We cannot rule out that some of the anti-D1R antibodies interacted with some epitopes on the D2R chain. If it is indeed the case then the cross reacting anti-D1R will be detected as anti-D2R as well. However, no correlation was found between the anti-D1R and the anti-D2R titers, and it seems unlikely that the “functional” anti-D2R antibodies are those which were generated against D1R.

Other antibodies such as anti-tubulin and anti-LGN were not significantly elevated suggesting that dopaminergic antibodies are specific and important components of an autoimmune process in SC In previous studies, both anti-tubulin and anti-lysoganglioside antibodies were significantly elevated when compared to our previously published control groups which excluded infections and inflammatory conditions and had lower ASLO titers.[5], [6], [7], [10] In our study herein, controls with infections and inflammatory conditions were used because the Ashkenazi population is susceptible to streptococcal infections throughout childhood. Thus, our controls used in our study are different from controls used previously and change the outcome of the data. However, even with the use of the controls with obvious streptococcal infections due to the elevated ASLO titers, the anti-D2R and anti-D1R antibodies were significantly elevated in the Sydenham chorea group. The lower titers of the anti-tubulin and anti-lysoganglioside may suggest that the antibodies against the D1R and D2R may be more responsible for the pathology seen in SC.

These antibody-targeted antigens namely, D1R and D2R, are key components in the regulation of the dopaminergic pathways which are considered to be the source of the chorea and behavioral symptoms. Consequently, both are successfully targeted with anti-dopaminergic drugs [14]. The association between chorea and the dopamine receptors has been implicated in animals and humans. The D1 receptor stimulation appears to be involved in the genesis of chorea in MPTP-treated squirrel monkeys [15]. D1 receptor agonists induce dyskinesia [16] while D2 receptors are known to be pathologically relevant in Huntington's disease [4], McLeod syndrome [17], neuroacanthocytosis [18] and parkinson associated dyskinesia [19].

Interestingly, we found that the ratio of the two autoantibody titers directed at D2R and D1R in SC correlated with clinical symptoms. In our study, the anti-D2R/anti-D1R antibody ratio correlated but moderately with USCRS symptoms r = 0.53 and non-motor symptoms r = 0.59. However, in 5 cases of Sydenham chorea who did not receive penicillin treatment, the total USCRS scores, non-motor scores, and the ratio of non-motor to motor scores were very strongly and positively correlated with anti-D2R antibody (r = 0.95, p = 0.05 for each of the symptom scores). Although this was a small sample, the correlation of Sydenham chorea symptoms with anti-D2R antibody was strikingly correlated at r = 0.95 with a p = 0.05.

Currently, we can only speculate but if the combined effect of these antibodies as manifested by the anti-D2R/D1R ratio does in fact correlate with the severity of clinical symptoms on the USCRS, it suggests that antibody-mediated activation/inhibition and/or modulation of the two different dopamine receptors may induce the clinical symptoms. The observed correlation between anti-D2R/D1R titers ratio may reflect the multifaceted physiologic interaction between the 2 dopaminergic receptors both intra and extracellularly. Intracellular cross-regulatory mechanisms between D1R- and D2R-activated signal transduction pathways act via a complex heterodimer formation [20], [21]. While extracellular activation of D1R on postsynaptic neurons has a moderate stimulatory effect on locomotion, the role of D2R which is expressed both presynaptically and postynaptically is more complex. Presynaptically localized autoreceptors generally provide an important negative feedback mechanism and postsynaptic receptors provide a positive stimulation for locomotor activity [22]. We propose that the main effect of anti-D2R with time may be down regulation of inhibitory presynaptic D2R autoreceptors thus causing an increase in dopamine release leading to excessive abnormal locomotor activity, presented as chorea. The effect of anti-D1R on psychiatric symptoms is unclear. Perhaps the relatively high level of anti-D1R antibodies downregulates D1R expression or alternatively blocks the interaction of dopamine with D1R. Thus at high anti-D1R titers the activation of this receptor by dopamine is attenuated leading to the suppression of excessive locomotive activity triggered by the anti-D2R antibodies. As a result, relatively high titers of anti-D1R antibodies may “protect” the child from the D2R induced chorea. Although we are unable to completely explain the combined effect of anti-D1R and anti-D2R on neuropsychiatric symptoms, there is a report associating low D1R binding of dopamine with obsessive compulsive disorder thus suggesting that low activity of D1R may be linked to behavioral symptoms such as those displayed in SC [23], [24]. In general, anti-D1R and anti-D2R autoantibodies may induce an imbalance within the dopaminergic system that results in greater sensitivity to dopamine stimulus.

In addition, in 4 children with SC who were not treated with penicillin, we found a correlation between chorea symptoms and anti-D2R antibody. This may provide indirect evidence that penicillin not only prevents further reinfection but may ameliorate chorea since the prolonged exposure to the bacteria in these children increases the exposure of their immune system to the bacterial antigens which ultimately lead to enhanced production of anti-neuronal autoantibodies and the chorea inducing ratio of anti D2R/D1R. Furthermore, the finding that anti-D2R titers correlate with ASLO strengthens the hypothesis that the streptococcal infection leads to anti-D2R antibody production ultimately causing clinical symptoms. Our data provide further support for the mimicry paradigm suggesting that autoantibodies, which are elevated following a streptococcal infection, recognize a structurally similar autoantigen, in this case an extracellular receptor, thus causing disease pathogenesis [5]. Evidence from cross-reactive human monoclonal antibodies derived from SC has supported the hypothesis that streptococcal antigens induce autoantibodies against neuronal antigens in the brain [5], [6]. Previous studies have shown that antibodies from SC interact with the basal ganglia [2], [25], [26], and antibodies in mice directed against group A beta-hemolytic streptococcal M protein have been shown to induce motor coordination deficits and changes in learning/memory and social behavior [27]. Moreover, studies in a rat model demonstrated motor disability and non-motor abnormalities associated with antibodies against the dopamine receptors following exposure to streptococcal antigens [7]. Our results are similar to those reported by Dale et al. In this recent study, 10/30 of the individuals with SC had elevated immunoglobulin-G to extracellular dopamine-2 receptor. These similar results are especially reinforced considering the different methodology (flow cytometry cell-based assays on HEK293 transfected cells), analysis (cut off >3 standard deviations above the control mean) and cohort (only 13/30 sera were common in both studies) [8].

In conclusion, our study supports the hypothesis that streptococcal infections lead to induction of anti-D1R and anti-D2R antibodies in SC and that the ratio between the titers of the two may determine the severity of symptoms in the disease. Although our sample size was small, we demonstrate an association between the ratio of antibody titers and clinically relevant data, suggesting a complex interaction between the two receptors which may lead to the motor and non-motor pathologies of SC. Our discovery that the anti-D2R/ D1R ratio rather than a single antibody titer correlated with neuropsychiatric symptoms may lead to a new way of understanding autoimmune neuropsychiatric disorders.
